# Association of Low-Attenuation Plaque with Impaired Glucose Tolerance and Type 2 Diabetes Mellitus in Patients with Suspected Coronary Artery Disease

**DOI:** 10.3390/biomedicines13010028

**Published:** 2024-12-26

**Authors:** Thomas R. Andersen, Katrine S. Overgaard, Laurits Juhl Heinsen, Roda Abdulkadir Mohamed, Helle Precht, Jess Lambrechtsen, Søren Auscher, Kenneth Egstrup

**Affiliations:** 1 Cardiovascular Research Unit, Odense University Hospital Svendborg, 5700 Svendborg, Denmark; katrine.schultz.overgaard@rsyd.dk (K.S.O.); laurits.juhl.heinsen@rsyd.dk (L.J.H.); roda.abdulkadir.mohamed@rsyd.dk (R.A.M.); hepr@ucl.dk (H.P.); jess.lambrechtsen@rsyd.dk (J.L.); soeren.auscher@rsyd.dk (S.A.); kenneth.egstrup@rsyd.dk (K.E.); 2 Department of Clinical Research, Faculty of Health Sciences, University of Southern Denmark, 5230 Odense, Denmark; 3 Health Sciences Research Centre, UCL University College, 5230 Odense, Denmark; 4 Radiology Department, Lillebelt Hospital, University Hospitals of Southern Denmark, 6000 Kolding, Denmark; 5 Institute of Regional Health Research, University of Southern Denmark, 5230 Odense, Denmark; 6 Discipline of Medical Imaging and Radiation Therapy, Adjunct to Cork University College, T12 EK59 Cork, Ireland

**Keywords:** computed coronary tomography angiography, low-attenuation plaque, dysglycemia, impaired glucose tolerance, type 2 diabetes mellitus, plaque burden, plaque composition

## Abstract

Aim: The aim of this study was to evaluate the differences in plaque composition and burden between normal glycemic status (NGS) and dysglycemia expressed as impaired glucose tolerance (IGT) and type 2 diabetes mellitus (T2DM). Methods: Clinically indicated coronary computed tomography angiography was used to evaluate patients with suspected coronary artery disease (CAD). An oral glucose tolerance test was performed to assess glycemic status. Patients were stratified as NGS, IGT, and T2DM. Plaque volumes were quantified using validated software, with further compositional measurements of low-attenuation, non-calcified, and calcified plaque burden. Results: Of 355 patients with suspected CAD, 220 had NGS, 92 were diagnosed with IGT, and 43 with known T2DM. Low-attenuation plaque volume was significantly higher in IGT (209 mm^3^, *p* < 0.02) and T2DM (243 mm^3^, *p* = 0.005) compared with NGS (166 mm^3^). Total plaque burden was similar between all groups, but a significantly greater low-attenuation plaque burden was seen in IGT (*p* = 0.03) and T2DM (*p* = 0.02) compared with NGS. The multivariate linear regression model adjusted for clinical risk factors showed that patients with IGT had a greater low-attenuation plaque burden compared with those with NGS (*p* = 0.03). Interestingly, no significant differences in plaque burdens were observed between those with IGT and T2DM in both univariate and multivariate analyses. Conclusions: Dysglycemia, including impaired glucose tolerance and type 2 diabetes mellitus, was associated with increased low-attenuation plaque burden compared with normal glycemic status. Patients with IGT demonstrated plaque burden similar to patients with known T2DM, underscoring the need for early metabolic intervention.

## 1. Introduction

Dysglycemia, characterized by perturbations in glucose metabolism, is steadily rising worldwide [1]. This condition develops gradually, progressing from normal glycemic status (NGS) to prediabetes (preDM) and, finally, type 2 diabetes mellitus (T2DM) [2]. The severity of dysglycemia correlates to higher morbidity and mortality after acute myocardial infarction [3,4], underscoring the importance of early detection and management.

Diabetes has long been a focus of coronary artery disease (CAD) [5] and can be diagnosed using either glycated hemoglobin A1c (HbA1c) or an oral glucose tolerance test (OGTT) [6]. Unlike HBA1c, the OGGT can stratify patients into prediabetic groups, impaired fasting glucose (IFG), and impaired glucose tolerance (IGT) based on their fasting glucose and 2 h values [6]. Independently, both IFG and IGT have been associated with CAD [7,8]. IGT has been more strongly associated with the risk of CAD and metabolic syndrome compared with IFG [9,10,11], which is why this article aims to highlight this association.

Patients with preDM often exhibit hallmarks of insulin resistance with increased inflammation, oxidative stress, and dyslipidemia [12]. These conditions are part of the latent phase of T2DM and are strongly associated with well-established risk factors for CAD, such as smoking, obesity, hypertension, and hypercholesterolemia [13]. Despite these associations, international guidelines for CAD risk management and prevention in patients with preDM remain noticeably absent. Interestingly, a study from Yahyavi et al. suggested that patients with HbA1c values of 46–47 mmol/mol, just below the limit for T2DM, face a higher risk of major adverse cardiovascular events (MACE) compared with those within the diabetic range [14]. This underscores the need for further research to investigate/clarify the mechanisms by which preDM influences CAD development, as well as determine the need for further risk prevention strategies.

Coronary computed tomography angiography (CCTA) is a valuable tool in screening for CAD and in risk stratification [15]. CCTA is a non-invasive imaging technique used to assess the extent of atherosclerosis and evaluate coronary artery stenosis. It also enables a comprehensive evaluation of plaque composition and allows for the estimation of plaque severity based on the plaque burden. Analyzing differences in attenuation allows for subdivision into different plaque components: calcified, non-calcified, and low-attenuation plaque burden [16,17]. Williams et al. found that non-calcified and low-attenuation plaque burden were predictors of both fatal and non-fatal myocardial infarction [17]. Research on low-attenuation plaque in patients with IGT is limited, and there is a significant gap in understanding the variations in low-attenuation plaque across the glycemic stages.

Our aim was to investigate the associations between plaque composition and burden in groups representing different stages of dysglycemia. Our objectives were (i) to evaluate the differences in plaque composition and burden between normal glycemic status, impaired glucose tolerance, and T2DM, and (ii) to evaluate the association between low-attenuation plaque burden and glycemic status, focusing on impaired glucose tolerance and its comparisons to normal glycemic status and T2DM.

## 2. Materials and Methods

### 2.1. Study Design

This was an observational, prospective, single-center, open-labeled study with a blinded comparison between glycemic status and coronary plaque composition and burden. The study was conducted at the Outpatient Clinic of Cardiology at OUH/ Svendborg Hospital, Denmark, and the Department of Cardiovascular Research. We enrolled patients referred for CCTA due to suspicion of ischemic heart disease between February 2018 and June 2020. During the CCTA visit, patients were informed about the study and invited to participate. Patients were booked for a consultation where written informed consent was acquired, blood samples were taken, and the completion of a medical history interview was conducted. The mean interval between blood sampling and CCTA was 29 days. Inclusion criteria included being at least 18 years old, being capable of providing informed consent, and having suspected CAD. Exclusion criteria included body mass index (BMI) > 40, irregular or fast heart rhythm inadequate for CCTA, reduced kidney function with estimated glomerular filtration rate (eGFR) < 45 mL/min, contrast allergy, or newly detected T2DM by either HbA1c or OGTT. All patients provided written informed consent, and the study was approved by the Regional Scientific Ethics Committee for Southern Denmark (project ID: S-20170094) and the Danish Data Protection Agency (project ID: 2012-58-0018).

### 2.2. Study Population

**Figure 1:**
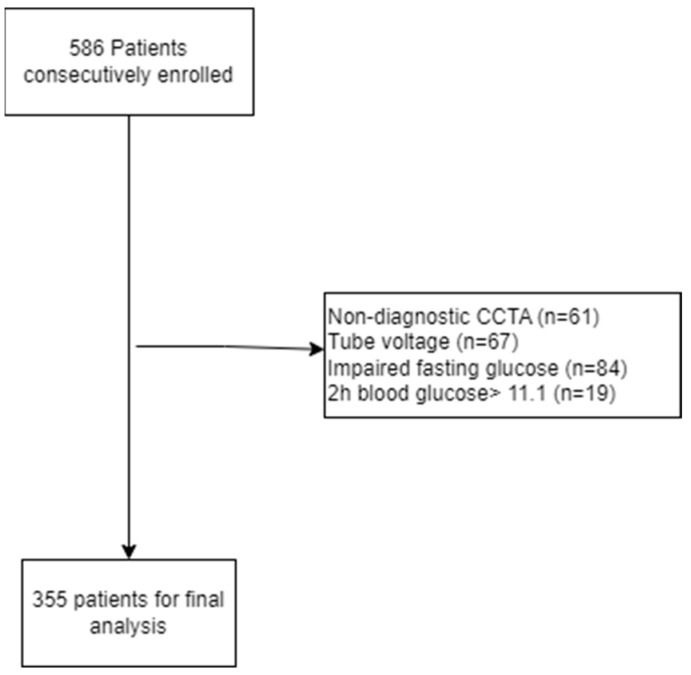
Consort diagram showing patient enrollment and exclusion.

For this study, 586 consecutive patients under suspicion of stable coronary artery disease CAD were enrolled in the Outpatient Clinic of Cardiology at OUH/Svendborg Hospital. Patients with non-diagnostic CCTA and tube voltage below 100 kilo Volt (kV) were excluded (n = 128) due to issues of comparability, as noted by Takagi et al. [18]. Eighty-four patients were excluded due to being stratified as IFG by OGTT. A further 19 patients were excluded based on 2 h blood glucose of more than 11.1 due to uncertainty of possible diabetes diagnosis. A total of 355 patients were available for the final analysis (Figure 1).

### 2.3. Biochemical Analysis

Blood samples were obtained and assessed for various biomarkers, including HbA1c, fasting blood glucose, 120 min blood glucose, total cholesterol, low-density lipoprotein (LDL), high-density lipoprotein (HDL), triglycerides, eGFR, creatinine, and C-reactive protein (CRP). Remnant cholesterol was calculated by subtracting LDL and HDL from total cholesterol.

### 2.4. Medical History

A questionnaire was used to evaluate cardiovascular risk factors, encompassing age, height, weight, smoking status, history of CAD, medical history, and medication use. Hypertension was defined by a previous diagnosis or current treatment with anti-hypertensive treatment. Hypercholesterolemia was defined by either previous diagnosis or current treatment with lipid-modifying drugs.

### 2.5. Oral Glucose Tolerance Test and HbA1c

**Figure 2:**
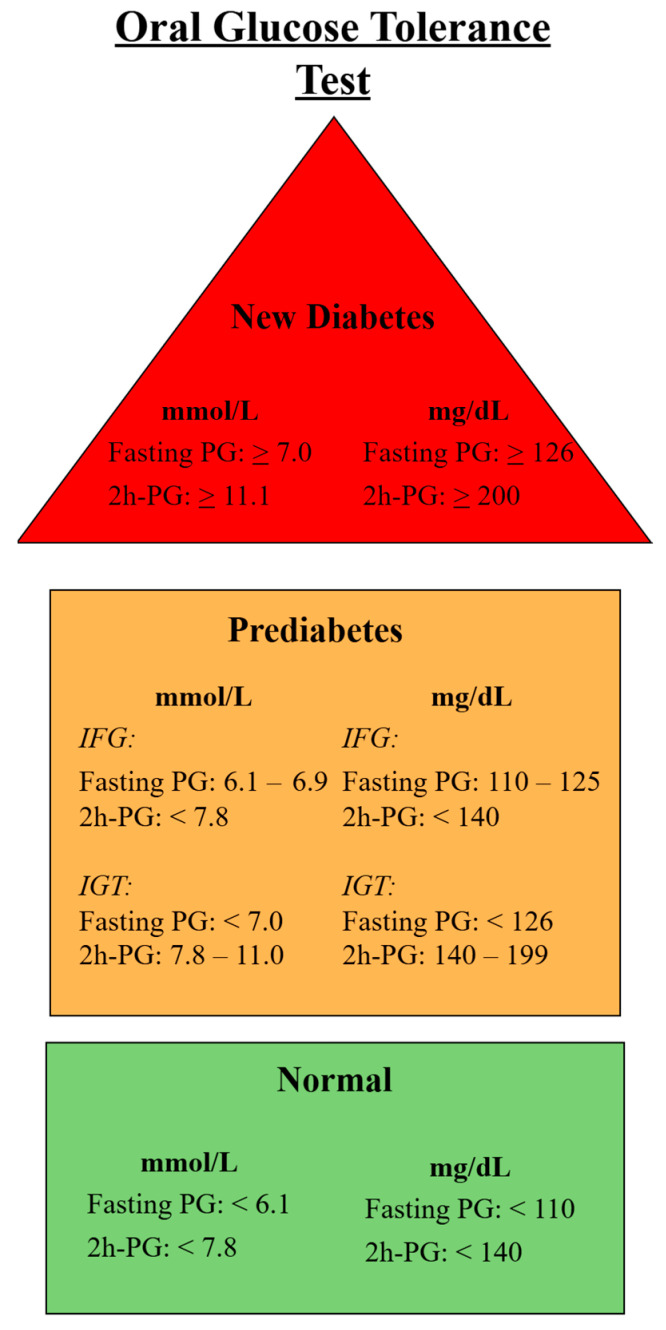
WHO criteria for oral glucose tolerance test stratification.

OGTT was performed after CCTA in patients without known T2DM. Patients were instructed to fast for 8 h before the OGTT. Fasting plasma glucose was measured before 75 g of glucose was ingested over five minutes. After 120 min, plasma glucose was measured, and patients were stratified according to WHO definitions of glycemic status (Figure 2) [6].

Patients with known T2DM were included in the study if they had an HbA1c ≥ 48 mmol/mol at inclusion or any prior two measurements, a previous venous plasma glucose of ≥11.1 mmol/L, or current use of antidiabetic medication. Patients were stratified into the following groups based on their OGTT results: NGS, IGT, and T2DM. Patients with IFG were excluded from the study due to similar risk profiles and plaque burden as the NGS group (Supplemental Material Tables S1 and S2).

### 2.6. Coronary Computed Tomography Angiography Acquisition

CCTA images were obtained using a standardized protocol on a 256-detector system (GE-revolution CT, GE Healthcare, Waukesha, WI, USA). We also conducted an unenhanced scan to assess coronary artery calcium. Ivabradine 7.5 mg tablets were administered one tablet on the night days before the scan and one tablet in the morning on scanning day to improve the image quality. If necessary, intravenous beta-blockers were administered on the day of the scan. ECG-gated prospective acquisition in the 75% R–R interval, with additional padding of 45 ms to allow for additional construction, was used to obtain the images. A total of 60 mL of iodine contrast (Visiplaque 320 mg iodine/mL) was injected at a rate of 5 mL/s, and the scan was timed when maximum attenuation was detected in the descending aorta. Tube voltage was adjusted based on the patient’s body size; patients scanned at 100–120 kV were included. The gantry rotation time was 280 ms with a 16 cm axial coverage. The slice thickness and interval for reconstruction were 0.625 mm, and 40% adaptive statistical iterative reconstruction was standardized. The highest-quality images were selected from the available phases and used for reconstruction and final analysis using dedicated software.

### 2.7. Quantitative Coronary Computed Tomography Angiography Analysis

Quantitative CCTA analysis was performed using the semiautomatic, validated software (Qangio CT Research Edition 3.2.0.13, Medis, Leiden NL, USA) [19]. All images were blinded to the experienced observer (TRA). The software automatically extracted the coronary artery tree and placed centerlines. Coronary artery segments were automatically extracted using the American Heart Association 17-segment model. Segments were manually changed when needed. Cross-sectional and longitudinal images of the lumen and vessel wall were automatically created, with careful manual adjustments when necessary. Segments with insufficient quality, with a lumen of less than 1.5 mm, or with low levels of contrast were excluded. Patients were excluded if they had less than two vessels of adequate quality or with severe artifacts that compromised image quality.

**Figure 3:**
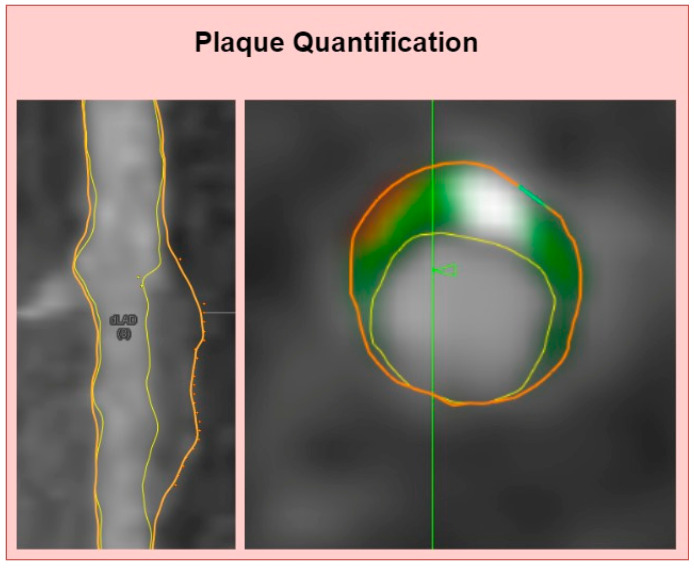
Representative longitudinal and transverse images of coronary plaque quantification on left anterior descending artery.

All vessels were examined for visible plaque, defined as structures ≥1 mm^3^ within or adjacent to the lumen and visible in ≥2 planes. Plaque volumes and burdens were extracted automatically per patient, and tissue volumes were extracted based on Hounsfield Unit (HU) values using a dynamic algorithm, converting HU thresholds into volumes according to luminal contrast densities. The following plaque characteristics were extracted by the software: vessel length (mm), lumen volume (mm^3^), and total atheroma volume (TAV, mm^3^). The total plaque volume was sub-classified into volumes of calcified plaque (≥351 HU), non-calcified plaque (30–350 HU), and low-attenuation plaque (<30 HU) based on their HU values (Figure 3). The following parameters were calculated post-data extraction: percent atheroma volume (PAV) ((total vessel volume − total lumen volume)/total vessel volume × 100%), normalized atheroma volume (NAV) ((total vessel volume − total lumen volume)/mean segment length), percent plaque volume per individual plaque component (PPV) ((total calcified, non-calcified and low-attenuation plaque volume/total plaque volume) × 100%). Additionally, patients were screened for the presence of significant stenosis (≥50% stenosis). The coronary artery calcium score (CACS) was calculated using the Agatston method on non-contrast CCTA images [20].

### 2.8. Statistical Analysis

All statistical analysis was performed using STATA IC 17. Baseline characteristics are presented as means with standard deviations for continuous variables, and categorical variables are presented as totals and percentages of the total number per group. Patient and plaque level data were tested for normal distribution prior to analysis. Non-normally distributed variables are presented as medians and inter-quartile ranges. Student’s *t*-test was used to compare the means of continuous variables between each pair of the three stratified groups: NGS vs. IGT, NGS vs. T2DM, and IGT vs. T2DM. When appropriate, categorical parameters were described using the Chi-squared or Fisher’s test. Plaque volumes were logarithmically transformed before statistical analysis due to their non-normal distribution. The association between NGS, IGT, and T2DM groups with overall plaque burden and individual plaque components was tested using univariate and multivariate linear regression. Categorical parameters, stenosis ≥ 50%, and plaque presence were tested using univariate and multivariate logistic regression. To build the multivariate regression model, baseline characteristics with a *p*-value < 0.05 in univariate analysis were initially considered for inclusion. The Wald Test was then applied to evaluate the significance of each variable within the multivariate context, assessing whether it significantly improved the model’s fit while controlling for other factors. Variables with *p*-values < 0.05 on the Wald Test were retained in the final model. Additionally, variables known to be clinically relevant confounders based on prior research or biological plausibility were included regardless of their univariate *p*-values.

### 2.9. Statement on Use of AI

We used ChatGPT, developed by OpenAI, to enhance the clarity and accuracy of the manuscript’s language. The platform was accessed from October to 20th of December 2024, and its functionality was employed specifically for refining the wording and improving the articulation of key concepts. The authors take full responsibility for the integrity and accuracy of the content generated and confirm that all substantive scientific findings and conclusions remain the work of the authors.

## 3. Results

### 3.1. Patient Population

This cross-sectional study included 355 patients under suspicion of CAD. Of these, 220 patients were classified as NGS, 92 patients as IGT, and 43 patients had known T2DM. The baseline characteristics of the study participants are presented in Table 1. The BMI was significantly higher in the IGT and T2DM groups compared with the NGS group (IGT: 29.3 ± 4.5, T2DM: 31.5 ± 6.0, and NGS: 27.0 ± 4.0). The IGT group had higher systolic blood pressure compared with both NGS and T2DM. The prevalence of hypercholesterolemia increased gradually across the spectrum of dysglycemia (i.e., NGS 25%, IGT 40%, T2DM 79%). The T2DM group was more likely to be treated with anti-hypertensive medications, and lipid-lowering agents compared with NGS and IGT groups. Additionally, the T2DM group had an increased risk of having a family history of CAD.

Glycemic status significantly influenced the lipid profiles and inflammatory markers, with distinct differences observed among the T2DM, IGT, and NGS groups. The total cholesterol and the low-density lipoprotein (LDL) levels were significantly lower in the T2DM group compared with the other groups (*p* < 0.001). Remnant lipoproteins were significantly higher in the IGT group compared with the NGS (*p* < 0.0001). Triglyceride levels increased as glycemic status worsened (NGS: 1.4 ± 0.9, IGT 1.8 ± 0.9, T2DM: 1.9 ± 1.1). Finally, the IGT group had a significantly higher level of C-reactive protein compared with the NGS (*p* < 0.0001) and T2DM groups (*p* = 0.006).

### 3.2. CCTA Findings

Table 2 presents the total atheroma volume and individual plaque volumes, as well as the prevalence of stenosis of more than 50% in the study groups. Total atheroma volume, calcified, and non-calcified plaque volume did not differ between the groups. However, the low-attenuation plaque volume was significantly higher in patients with IGT and T2DM compared with patients with NGS. Specifically, the total volume of low-attenuation plaque was significantly higher in the IGT and T2DM groups compared with NGS (*p* < 0.001) (Table 2). Notably, no difference was observed between the IGT and T2DM groups. A significant trend was found for the increasing number of significant stenosis present as dysglycemia worsened. However, there was no statistically significant difference between NGS and IGT.

### 3.3. Univariate and Multivariate Analysis

Table 3 presents a comparative analysis of plaque volume and burden, CACS, and the presence of stenosis of more than 50%. In the unadjusted model, a higher burden of low-attenuation plaque was associated with dysglycemia. Patients with IGT and T2DM had a significantly higher burden of low-attenuation plaques compared to patients with NGS. However, no difference was found between patients with IGT compared to patients with T2DM. The presence of stenosis of more than 50% was associated with having T2DM. A multivariate regression analysis was performed to account for possible confounders. The following parameters were tested: sex, age, LDL, hypercholesterolemia, smoking, BMI, and CRP. The analysis revealed that low-attenuation plaque burden remained significantly associated with IGT and T2DM when compared with NGS.

## 4. Discussion

In this cross-sectional study, we investigated the associations between plaque composition and burden in groups representing different stages of dysglycemic progression. Our findings indicate the following: (i) Plaque composition varies significantly, with a significant trend of incremental increase in non-calcified and low-attenuation plaque observed moving from normal glycemic status to impaired glucose tolerance and type 2 diabetes. However, the total plaque burden did not vary across groups. (ii) Patients with IGT and T2DM showed a higher burden of low-attenuation plaque compared to those classified as NGS.

### 4.1. Dysglycemia and CAD

Type 2 diabetes mellitus is a well-recognized risk factor for plaque accumulation and the development of CAD [21,22,23]. In contrast, the impact of preDM, expressed as IFG and IGT, is not as clear [11]. Previous studies have shown inconsistent results regarding their influence on atherosclerosis. Some report a significant association, whereas others found no consistent relationship between CAD and IGT or IFG [5,10,24,25]. This inconsistency has been further complicated by the multitude of ways to classify preDM. HbA1c has become the gold standard due to accessibility and because of the methodological impracticalities of OGTT. However, using HbA1c for diagnosing T2DM and preDM has been shown to underestimate the incidence of both conditions, and thus, this practice might misclassify patients at elevated risk of CAD [13].

In our study, we used OGTT as the stratification method to detect dysglycemia, as this allows for the earliest possible detection of disturbances in blood glucose levels. Furthermore, the OGTT is the only way to obtain the IGT population. OGTT detects both IFG and IGT, but since IGT in previous reports has been strongest associated with CAD, we chose to focus on this population in the present study. For transparency, data on the IFG population have been included in Supplementary Materials. We found that stratifying patients using HbA1c values was not associated with plaque burden. This was most likely due to a lack of statistical power since we did not have a large group of patients with prediabetic HbA1c values.

### 4.2. Plaque Burden Across Glycemic Control

Total plaque burden has been associated with an increased risk of CAD and MACE in previous reports [22], and a trend of increasing plaque volume with worsening dysglycemia has been observed in previous studies [26,27,28]. In our study, which used OGTT as a stratification tool, we found no significant difference in the total plaque burden between the groups. To the best of our knowledge, this is the first CCTA study to find a similar trend for dysglycemia and establish T2DM in relation to total plaque volume. Previous CCTA studies found a higher total plaque burden in patients with T2DM compared with IGT/preDM, which we did not observe in our data [21,24,29]. This may be attributed to the good glycemic control in our diabetes cohort, with a mean HbA1c of 52 mmol/mol. Kim et al. showed that plaque progression was halted with reductions in HbA1c [22], which might explain our findings in our cohort of well-regulated patients with T2DM. Additionally, we found elevated CRP levels in the IGT group compared to NGS and T2DM. While CRP is a well-established marker of systemic inflammation, it did not correlate with total plaque burden in our study, aligning with evidence that CRP alone does not independently predict total plaque burden [30]. However, the elevated CRP in the IGT group may indicate a distinct inflammatory profile at this intermediate glycemic stage, potentially influencing plaque vulnerability rather than overall burden. Limited research exists on low-attenuation plaque in IGT patients, leaving a significant gap in understanding variations in plaque characteristics across the glycemic stage.

### 4.3. Trends of Low-Attenuation Plaque, CACS, and Dysglycemia

The importance of coronary artery plaque composition cannot be understated. Studies have shown correlations between the presence of low-attenuation plaque and an increased risk of myocardial death [17,31]. In our study, we found a trend of increasing low-attenuation plaque burden by worsening degree of dysglycemia. The same trend was observed in a cohort study using fasting glucose or HbA1c [21]. IGT and T2DM share key metabolic pathways, including insulin resistance, hyperglycemia-induced oxidative stress, and chronic inflammation [32]. These mechanisms are associated with the development of atherosclerosis [33], which may explain the similar plaque compositions observed in our study between the IGT and T2DM groups. To the best of our knowledge, this is the first time such an association has been identified using IGT as a measure of preDM.

Furthermore, we found that CACS showed a significant trend with worsening dysglycemia. CACS is known to be higher in diabetic populations and associated with dysglycemia [23]. In our cohort, this may also be attributed to higher usage of statins in the T2DM and IGT groups, as statins are known to promote calcification [34].

### 4.4. Prognosis and Lack of Guidelines

Patients with preDM, despite the classification method, have an increased risk of CAD compared with NGS [11,14]. Currently, the guidelines for risk prevention in patients with preDM are loosely based on T2DM guidelines [35], but dedicated preDM guidelines are absent. In a Danish cohort, Yahyavi et al. found that patients with sub-diabetic HbA1c levels had a higher risk of major cardiovascular events compared with patients with T2DM with excellent glycemic control. A meta-analysis of early intervention studies for patients with preDM found that early interventions lowered all-cause mortality by up to 17% [36]. However, there was no consensus on medicinal interventions, as most studies focused primarily on exercise and dietary interventions. In our cohort, patients with IGT were less medicated and had higher LDL-cholesterol and inflammatory biomarkers compared with patients with T2DM and NGS.

## 5. Limitations

This study has several limitations. Since it is an observational study, we cannot establish causality between dysglycemia and unfavorable plaque composition and burden. Patients were invited to join the study after CCTA had been conducted, resulting in blood samples not being taken on the same day. There is daily variability in several metabolic markers used in this study, which could lead to some inaccuracy. However, this delay was present for all patients, thus negating some of the possible errors. Using OGTT as a stratification tool allows for the early detection of dysglycemia, but it relies on a single measurement of blood glucose and is prone to variability. The uneven distribution of patients across the three groups could potentially account for the observed similarity in plaque burdens between the IGT and T2DM groups while also affecting the statistical power and reproducibility of the findings. We only used one software program for the analysis of CCTA data, and it could have been beneficial to compare two methodologies to avoid any inaccuracies. The software used for this study has been validated and is semi-automated, there is still some manual assessment of plaque, which could lead to uncertainties.

## 6. Conclusions

In this observational study, we found an increased burden of low-attenuation plaque in patients with impaired glucose tolerance and type 2 diabetes mellitus compared with normal glycemic status. This incremental increase could explain the high incidence of CAD in patients with prediabetes. Further research is needed to understand plaque progression in prediabetic states, explore targeted interventions, and confirm the clinical relevance of low-attenuation plaque burden in predicting adverse outcomes.

## Figures and Tables

**Table 1:** Patient characteristics. Data are presented as mean ± SD or as exact figure (n) with percentage (%).

	NGS N = 220	IGT N = 92	T2DM N = 43	NGS vs. IGT *p*-Value	NGS vs. T2DM *p*-Value	IGT vs. T2DM *p*-Value
Age	59.8 (11.7)	61.6 (10.6)	62.8 (9.3)	0.5	0.3	0.9
Male gender	112 (51%)	57 (62%)	29 (67%)	0.07	0.05	0.6
BMI	27.0 (4.0)	29.3 (4.5)	31.5 (6.0)	<0.0001	<0.0001	0.02
Systolic BP	140 (22)	149 (20)	140 (22)	0.004	0.9	0.1
Diastolic BP	78 (11)	82 (13)	80 (17)	0.05	0.9	0.9
Hypertension	79 (36%)	45 (49%)	27 (63%)	0.03	0.001	0.1
Hypercholesterolaemia	54 (25%)	37 (40%)	34 (79%)	0.005	<0.0001	<0.0001
Never smoker	110 (50%)	33 (36%)	18 (42%)	0.02	0.3	0.5
Former smoker	82 (37%)	45 (49%)	20 (47%)	0.06	0.6	0.6
Active smoker	28 (13%)	14 (15%)	5 (12%)	0.6	0.8	0.6
Fam. history of CVD	71 (33%)	32 (35%)	22 (51%)	0.7	0.02	0.08
Fasting glucose	5.6 (0.3)	6.1 (0.5)	-	<0.0001	-	-
120 min glucose	5.8 (1.1)	9.0 (0.9)	-	<0.0001	-	-
HbA1c	35 (4)	37 (3)	52 (11)	0.008	<0.0001	<0.0001
Total cholesterol	5.0 (1.0)	4.7 (1.1)	3.6 (0.8)	0.08	<0.0001	<0.0001
HDL	1.5 (0.5)	1.4 (0.4)	1.1 (0.2)	0.002	<0.0001	0.02
LDL	3.0 (0.9)	2.6 (1.0)	1.9 (0.5)	0.01	<0.0001	<0.0001
Triglycerides	1.4 (0.9)	1.8 (0.9)	1.9 (1.1)	0.001	0.002	0.9
Remnant lipoprotein	0.5 (0.4)	0.7 (0.4)	0.6 (0.4)	<0.0001	0.9	0.2
CRP	2.1 (2.0)	3.3 (2.9)	2 (2.0)	<0.0001	0.9	0.006
eGFR	81 (10)	82 (10)	82 (11)	0.9	0.9	0.9
Creatinine	77 (16)	78 (16)	76 (15)	0.9	0.9	0.9
ACE-inhibitors	42 (19%)	28 (30%)	24 (56%)	0.03	<0.0001	0.005
Calcium-antagonists	28 (13%)	16 (17%)	11 (26%)	0.3	0.03	0.3
Beta-blockers	40 (18%)	23 (25%)	15 (35%)	0.2	0.01	0.2

**Table 2:** Plaque burden and composition stratified by glycemic state in 355 patients. Data are presented as mean and SD when data is continuous. Stenosis is presented as the total number in the group and percentage. Total CAC is presented as median and IQR.

	NGS N = 220	IGT N = 92	T2DM N = 43	NGS vs. IGT *p*-Value	NGS vs. T2DM *p*-Value	IGT vs. T2DM *p*-Value	Trend *p*-Value
TAV, mm^3^	955 (786–1172)	1054 (873–1294)	1077 (875–1317)	0.3	0.4	0.9	0.1
PAV, %	27 (23–30)	27 (23–31)	27 (24–32)	0.9	0.8	0.9	0.5
NAV, %	2.7 (2.3–3.4)	2.9 (2.3–3.6)	2.9 (2.3–3.5)	0.8	0.9	0.9	0.4
Calcified plaque vol.	25 (9–87)	43 (7–119)	50 (21–112)	0.8	0.2	0.3	0.1
Non-calcified vol.	728 (607–866)	764 (616–905)	728 (611–933)	0.9	0.8	0.9	0.7
Low-attenuation vol.	166 (112–230)	209 (154–306)	243 (145–350)	0.02	0.001	0.9	<0.0001
PPV calcified	3 (1–8)	4 (1–10)	5 (2–11)	0.9	0.4	0.3	0.2
PPV non-calcified	76 (70–82)	71 (66–78)	70 (65–75)	0.04	0.005	0.8	0.01
PPV low-attenuation	16 (13–23)	19 (14–28)	22 ( (16–28)	0.03	0.02	0.9	0.004
Stenosis > 50%	20 (9%)	14 (15%)	13 (30%)	0.1	0.001	0.005	0.0002
Total CAC score	8 (0–106)	29 (1–214)	84 (13–266)	0.05	0.5	0.9	0.05

TAV: total atheroma volume, PAV: percent atheroma volume, NAV: normalized atheroma volume, PPV: percentage plaque volume, NGS: normal glycemic status. IGT: prediabetes. T2DM: type 2 diabetes mellitus. Stenosis > 50%: Stenosis of coronary artery greater than 50% of luminal area. Total CAC: total coronary artery calcium score.

**Table 3:** Univariate and multivariate linear and logistic regression analysis of plaque volumes, total coronary artery calcium score, and luminal stenosis of more than 50%. Multivariate model was adjusted for sex, age, LDL, hypercholesterolemia, smoking, BMI, and CRP. OR: odds ratio.

	NGS vs. IGT β	*p*-Value	NGS vs. T2DM β	*p*-Value	IGT vs. T2DM β	*p*-Value
Unadjusted model						
Calcified PPV	−0.01	0.7	0.18	0.1	0.4	0.1
Non-calcified PPV	−0.04	0.02	−0.03	0.002	−0.03	0.2
Low–attenuation PPV	0.14	0.01	0.12	0.007	0.08	0.3
Stenosis > 50%	1.8	0.07	2.1	<0.0001	2.4	0.05
Adjusted model						
Calcified PPV	−0.09	0.6	0.13	0.3	0.20	0.5
Non-calcified PPV	−0.02	0.1	−0.02	0.08	−0.01	0.5
Low-attenuation PPV	0.12	0.02	0.09	0.02	0.07	0.4
Stenosis > 50%	1.3	0.5	2.7	0.03	1.8	0.2

## Data Availability

The data presented in this study are available on request from the corresponding author.

## Supplementary Materials

The following supporting information can be downloaded at https://www.mdpi.com/article/10.3390/biomedicines13010028/s1, Table S1 IFG vs. NGS baseline; Table S2 IFGvsNGS univariate and multivariate analysis.
