# Supplementary material for: Association of Low-Attenuation Plaque with Impaired Glucose Tolerance and Type 2 Diabetes Mellitus in Patients with Suspected Coronary Artery Disease
Source: Biomedicines. 2024 Dec 26;13(1):28. doi: 10.3390/biomedicines13010028 (PMC11760424; doi:10.3390/biomedicines13010028)
Supplement: Supplementary file 1 [file biomedicines-13-00028-s001.zip › biomedicines-3349994-supplementary.pdf]

biomedicines

MDPI

Table S1. Showing patient characteristics for NGS and IFG patients. Data given as mean±SD or as exact figure (n) with percentage (%).

	NGS<br>N = 220	IFG<br>N = 84	*p*-value
Age	59.8(11.7)	59.9(10.8)	0.8
Gender male	112(51%)	59(70%)	**0.003**
BMI	27.0(4.0)	27.4(3.6)	0.3
Systolic BP	140(22)	142(14.9)	0.1
Diastolic BP	78(11)	80.4(8.6)	0.06
Hypertension	79(36%)	24(29%)	0.1
Hypercholesterolaemia	54(25%)	21(25%)	0.9
Never smoker	110(50%)	33(39%)	**0.1**
Former smoker	82(37%)	37(44%)	0.2
Active smoker	28(13%)	14(17%)	0.4
Fam. History of CVD	71(33%)	29(35%)	0.7
Fasting glucose	5.6(0.3)	6.3(0.3)	**< 0.0001**
120min glucose	5.8(1.1)	6.2(1.0)	**0.003**
HbA1c	35(4)	36(3)	0.07
Total cholesterol	5.0(1.0)	5.0(1.0)	0.8
HDL	1.5(0.5)	1.4(0.4)	0.06
LDL	3.0(0.9)	3.0(0.9)	0.8
Triglycerides	1.4(0.9)	1.5(0.8)	0.08
Remnant lipoprotein	0.5(0.4)	0.6(0.4)	0.2
CRP	2.1(2.0)	2.2(1.9)	0.5
eGFR	81(10)	81(10)	0.6
Creatinine	77(16)	82(14.8)	**0.03**
ACE-inhibitors	42(19%)	21(25%)	0.4
Calcium-antagonists	28(13%)	9(11%)	0.5
Beta-blockers	40(18%)	13(16%)	0.6

Table S2. Plaque burden and composition for NGS and IFG. Data are presented as mean and SD when data is continuous. Stenosis is presented as the total number in the group and percentage. Total CAC is presented as median and IQR. TAV: total atheroma volume, PAV: percent atheroma volume, NAV: normalised atheroma volume, PPV: percentage plaque volume, NGS: normal glycemic status. IFG: Impaired fasting glucose. T2DM: type 2 diabetes mellitus. Stenosis > 50%: Stenosis of coronary artery greater than 50% of luminal area. Total CAC: total coronary artery calcium score.

	NGS<br>N = 220	IFG<br>N = 84	*p*-value
**TAV, mm³**	955(786-1172)	865(664-1025)	0.6
**PAV, %**	27(23-30)	26.5(23-29)	0.5
**NAV, %**	2.7(2.3-3.4)	3(2.3-3.5)	0.6
**Calcified plaque vol.**	25(9-87)	33(9-67)	0.6
**Non-calcified vol.**	728(607-866)	789(643-931)	0.2
**Low attenuation vol.**	166 (112-230)	174(131-238)	0.5
**PPV calcified**	3(1-8)	4(1-8)	0.4
**PPV non-calcified**	76(70-82)	78(71-84)	0.3
**PPV Low-attenuation**	16(13-23)	17(15-25)	0.3
**Stenosis > 50%**	20(9%)	9(10%)	0.9
**Total CAC score**	8(0-106)	17(0-134)	0.6

 https://doi.org/10.3390/biomedicines13010028
